# Supplementary material for: Evaluating the African food supply against the nutrient intake goals set for preventing diet-related non-communicable diseases: 1990 to 2017 trend analysis
Source: PLoS One. 2021 Jan 11;16(1):e0245241. doi: 10.1371/journal.pone.0245241 (PMC7799762; doi:10.1371/journal.pone.0245241)
Supplement: Attachment — Submitted filename: PONE-D-20-31495_reviewer comments.pdf [file pone.0245241.s006.pdf]

PLOS ONE

Evaluating the African food supply against the nutrient intake goals set for preventing diet-related non-communicable diseases: 1990 to 2017 trend analysis

--Manuscript Draft--

**Manuscript Number:**	PONE-D-20-31495
**Article Type:**	Research Article
**Full Title:**	Evaluating the African food supply against the nutrient intake goals set for preventing diet-related non-communicable diseases: 1990 to 2017 trend analysis
**Short Title:**	Nutrient intake goals for preventing non-communicable diseases in Africa.
**Corresponding Author:**	Samson Gebremedhin, PhD<br>Addis Ababa University<br>Addis Ababa, ETHIOPIA
**Keywords:**	Dietary risk factors; Non-communicable diseases; Food supply; Food balance sheet; Africa
**Abstract:**	Background<br><br>Population intake goals intended to prevent diet-related non-communicable diseases (NCDs) had been set for multiple nutrients. Yet, little is known whether the existing food supply in Africa is in conformation with these goals or not. We evaluated the African food balances against the recommendations for macronutrients, free sugars, types of fatty acids, cholesterol and fruits and vegetables over 1990 to 2017, and provided regional, sub-regional and country-level estimates.<br><br>Methods<br><br>The per capita supply of 95 food commodities for 45 African countries (1990-2017) was accessed from the FAOSTAT database and converted to calories, carbohydrate, fat, protein, free sugars, cholesterol, saturated (SFA), monounsaturated (MUFA), and polyunsaturated (PUFA) fatty acids contents using the Food Data Central database. The supply of fruits and vegetables was also computed.<br><br>Results<br><br>In Africa the energy supply ~~was~~ increased by 16.6% from 2,685 in 1990 to 3,132 kcal/person/day in 2017. However, the energy contribution of carbohydrate, fat and protein remained constant and almost within acceptable range around 73, 10 and 9%, respectively. In 2017, energy from fats surpassed the 20% limit in upper-middle- or high-income and Southern Africa countries. Energy from SFA remained within range (<10%) but that of PUFA was below the minimum desirable level of 6% in 28 countries. Over the period, energy from free sugars remained constant around 7% but the figure exceeded the limit of 10% in upper-middle- or high-income countries (14.7%) and in Southern (14.8%) and Northern (10.5%) sub-regions. Between 1990 and 2017 the availability of dietary cholesterol per person surged by 14% but was below the limit of 300 mg/day. The supply of fruits and vegetables increased by 27.5% from 279 to 356 g/capita/day; yet, with the exception of ~~the~~ Northern Africa, the figure remained below the target of 400 g/capita/day in all sub-regions.<br><br>Conclusion<br><br>In African most population intake goals within acceptable range. Yet, the supplies of fruits and vegetables and PUFAs are suboptimal and the increasing energy contributions of free sugars and fats are emerging concerns in specific sub-regions.
**Order of Authors:**	Samson Gebremedhin, PhD
	Tilahun Bekele
**Additional Information:**	

Powered by Editorial Manager® and ProduXion Manager® from Aries Systems Corporation

Question	Response
**Financial Disclosure**<br><br>Enter a financial disclosure statement that describes the sources of funding for the work included in this submission. Review the submission guidelines for detailed requirements. View published research articles from *PLOS ONE* for specific examples.<br><br>This statement is required for submission and **will appear in the published article** if the submission is accepted. Please make sure it is accurate.<br><br>**Unfunded studies**<br>Enter: *The author(s) received no specific funding for this work.*<br><br>**Funded studies**<br>Enter a statement with the following details:<br>• Initials of the authors who received each award<br>• Grant numbers awarded to each author<br>• The full name of each funder<br>• URL of each funder website<br>• Did the sponsors or funders play any role in the study design, data collection and analysis, decision to publish, or preparation of the manuscript?<br>• **NO** - Include this sentence at the end of your statement: *The funders had no role in study design, data collection and analysis, decision to publish, or preparation of the manuscript.*<br>• **YES** - Specify the role(s) played.<br><br>* typeset	The authors received no specific funding for this work.
**Competing Interests**<br><br>Use the instructions below to enter a competing interest statement for this submission. On behalf of all authors, disclose any competing interests that could be perceived to bias this work—acknowledging all financial support and any other relevant financial or non-financial competing interests.<br><br>This statement **will appear in the published article** if the submission is	The authors have declared that no competing interests exist.

accepted. Please make sure it is accurate. View published research articles from *PLOS ONE* for specific examples.<br><br>**NO authors have competing interests**<br><br>Enter: *The authors have declared that no competing interests exist.*<br><br>**Authors with competing interests**<br><br>Enter competing interest details beginning with this statement:<br><br>*I have read the journal's policy and the authors of this manuscript have the following competing interests: [insert competing interests here]*<br><br>* typeset	
**Ethics Statement**<br><br>Enter an ethics statement for this submission. This statement is required if the study involved:<br><br>• Human participants<br>• Human specimens or tissue<br>• Vertebrate animals or cephalopods<br>• Vertebrate embryos or tissues<br>• Field research<br><br>Write "N/A" if the submission does not require an ethics statement.<br><br>General guidance is provided below. Consult the submission guidelines for detailed instructions. **Make sure that all information entered here is included in the Methods section of the manuscript.**	N/A

**Format for specific study types**<br><br>**Human Subject Research (involving human participants and/or tissue)**<br>• Give the name of the institutional review board or ethics committee that approved the study<br>• Include the approval number and/or a statement indicating approval of this research<br>• Indicate the form of consent obtained (written/oral) or the reason that consent was not obtained (e.g. the data were analyzed anonymously)<br><br>**Animal Research (involving vertebrate animals, embryos or tissues)**<br>• Provide the name of the Institutional Animal Care and Use Committee (IACUC) or other relevant ethics board that reviewed the study protocol, and indicate whether they approved this research or granted a formal waiver of ethical approval<br>• Include an approval number if one was obtained<br>• If the study involved *non-human primates*, add *additional details* about animal welfare and steps taken to ameliorate suffering<br>• If anesthesia, euthanasia, or any kind of animal sacrifice is part of the study, include briefly which substances and/or methods were applied<br><br>**Field Research**<br><br>Include the following details if this study involves the collection of plant, animal, or other materials from a natural setting:<br>• Field permit number<br>• Name of the institution or relevant body that granted permission	
**Data Availability**<br><br>Authors are required to make all data underlying the findings described fully available, without restriction, and from the time of publication. PLOS allows rare exceptions to address legal and ethical concerns. See the PLOS Data Policy and FAQ for detailed information.	Yes - all data are fully available without restriction

A Data Availability Statement describing where the data can be found is required at submission. Your answers to this question constitute the Data Availability Statement and **will be published in the article**, if accepted.<br><br>**Important:** Stating 'data available on request from the author' is not sufficient. If your data are only available upon request, select 'No' for the first question and explain your exceptional situation in the text box.<br><br>Do the authors confirm that all data underlying the findings described in their manuscript are fully available without restriction?	
**Describe where the data may be found in full sentences. If you are copying our sample text, replace any instances of XXX with the appropriate details.**<br><br>• If the data are **held or will be held in a public repository**, include URLs, accession numbers or DOIs. If this information will only be available after acceptance, indicate this by ticking the box below. For example: *All XXX files are available from the XXX database (accession number(s) XXX, XXX.*).<br>• If the data are all contained **within the manuscript and/or Supporting Information files**, enter the following: *All relevant data are within the manuscript and its Supporting Information files.*<br>• If neither of these applies but you are able to provide **details of access elsewhere**, with or without limitations, please do so. For example:<br><br>*Data cannot be shared publicly because of [XXX]. Data are available from the XXX Institutional Data Access / Ethics Committee (contact via XXX) for researchers who meet the criteria for access to confidential data.*<br><br>*The data underlying the results presented in the study are available from (include the name of the third party*	Data are freely available from the FAOSTAT database. Please use the following links to access the data:<br>http://www.fao.org/faostat/en/#data/FBSH<br>http://www.fao.org/faostat/en/#data/FBS

Powered by Editorial Manager® and ProduXion Manager® from Aries Systems Corporation

and contact information or URL).<br>• This text is appropriate if the data are owned by a third party and authors do not have permission to share the data.<br><br>* typeset	
Additional data availability information:	

Manuscript

Click here to access/download;Manuscript;NCD and food supply manuscript.docx

Evaluating the African food supply against the nutrient intake goals set for preventing diet-related non-communicable diseases: 1990 to 2017 trend analysis

Samson Gebremedhin[1*], Tilahun Bekele[2]

[1]School of Public Health, Addis Ababa University, Addis Ababa, Ethiopia.

[2]Center for Food Science and Nutrition, Addis Ababa University, Addis Ababa, Ethiopia.

[*] Corresponding author

E-mail: samsongmgs@yahoo.com

Short title: Nutrient intake goals for preventing non-communicable diseases in Africa.

Abstract

Background: Population intake goals intended to prevent diet-related non-communicable diseases (NCDs) had been set for multiple nutrients. Yet, little is known whether the existing food supply in Africa is in conformation with these goals or not. We evaluated the African food balances against the recommendations for macronutrients, free sugars, types of fatty acids, cholesterol and fruits and vegetables over 1990 to 2017, and provided regional, sub-regional and country-level estimates.

Methods: The per capita supply of 95 food commodities for 45 African countries (1990-2017) was accessed from the FAOSTAT database and converted to calories, carbohydrate, fat, protein, free sugars, cholesterol, saturated (SFA), monounsaturated (MUFA), and polyunsaturated (PUFA) fatty acids contents using the Food Data Central database. The supply of fruits and vegetables was also computed.

Results: In Africa the energy supply was increased by 16.6% from 2,685 in 1990 to 3,132 kcal/person/day in 2017. However, the energy contribution of carbohydrate, fat and protein remained constant and almost within acceptable range around 73, 10 and 9%, respectively. In 2017, energy from fats surpassed the 20% limit in upper-middle- or high-income and Southern Africa countries. Energy from SFA remained within range (<10%) but that of PUFA was below the minimum desirable level of 6% in 28 countries. Over the period, energy from free sugars remained constant around 7% but the figure exceeded the limit of 10% in upper-middle- or high-income countries (14.7%) and in Southern (14.8%) and Northern (10.5%) sub-regions. Between 1990 and 2017 the availability of dietary cholesterol per person surged by 14% but was below the limit of 300 mg/day. The supply of fruits and vegetables increased by 27.5% from 279 to 356 g/capita/day; yet, with the exception of the Northern Africa, the figure remained below the target of 400 g/capita/day in all sub-regions.

Conclusion: In African most population intake goals within acceptable range. Yet, the supplies of fruits and vegetables and PUFAs are suboptimal and the increasing energy contributions of free sugars and fats are emerging concerns in specific sub-regions.

Keywords: Dietary risk factors; Non-communicable diseases; Food supply; Food balance sheet; Africa.

Background

Every year 41 million deaths, equivalent to 71% of the total deaths, occur globally due to non-communicable diseases (NCDs) [1]. Between 1990 and 2017, the number of avertable NCD-related deaths globally increased by nearly 50% [2]. Specifically, cardiovascular diseases, cancers, chronic respiratory diseases and diabetes contribute to more than 80% of the total NCD-related deaths [1]. With the existing trends, the Sustainable Development Goals (SDGs) target to reduce pre-mature mortality from NCDs by one-third is unlikely to be realized [3].

NCDs once considered as diseases of affluence are now disproportionately affecting low- and middle-income countries (LMIC) [4]. The risk of death from NCDs is now higher in LMIC than high-income countries and more than 80% of the global NCD-related deaths now occur in LMIC [2,4]. According to the recent estimate of the World Health Organization (WHO), in Africa 41% of the total deaths are from NCDs and probability of premature death from the four major NCDs is 21% [6]. Furthermore, NCDs explain more than half of the total mortality in 11 of the 54 African nations [5]. In sub-Saharan Africa, between 1990 and 2017, disability adjusted life years attributable to NCDs has increased by 67% [6].

NCDs have multiple genetic, environmental and behavioural determinants. Yet, the epidemiological shift observed in the last few decades is primarily attributable to changes in few major modifiable risk factors including dietary factors [1,7]. A large body of evidence confirms that the rise in unhealthy diets is among the major drivers of the global NCD pandemic [7-10]. In 2017, 11 million adulthood deaths were attributable to dietary factors [7]. Inadequate intake of fruits, vegetables and dietary fibre and high intake of salt, sugar, alcohol and fats lead to NCDs [7-10]. Even within the domain of fatty acids, individual types have

distinct physiological properties: high intakes of saturated (SFA) and trans (TFAs) fatty acids are strongly linked with dyslipidaemia while optimal intake of polyunsaturated (PUFAs) and monounsaturated (MUFA) fatty acids has beneficial effects [8,11,12].

In 2003 WHO and Food and Agriculture Organization of the United Nations (FAO) proposed population intake goals for preventing NCDs [8]. According to the recommendation, optimal macronutrient intake ranges based on contribution to daily energy intake are 55-75% for carbohydrate, 15–30% for fat and 7–20% for protein. Calories from SFAs and TFAs have to be below 10 and 1% of the total intake, respectively and that of PUFAs should be within 6 to 10%. Further, energy derived from free sugars should not exceed 10%. Dietary cholesterol intake should be limited (< 300 mg/day) and adequate (> 400 g/day) ~~amount~~ of fruits and vegetables ~~has to be taken~~. Specific goals have been set for sodium chloride, total dietary fibre and ω-3 and ω-6 fatty acids as well [8].

In Africa where there is active rise in NCD-related mortality, limited information is available whether the existing food supply is in ~~conformation~~ with these goals or not. The current study evaluated the African food balances against population intake recommendations set for macronutrients, free sugars, fatty acids, dietary cholesterol and fruits and vegetables, assessed trends over three decades (1990- 2017), and provided regional (continental), sub-regional (geographic and gross national income classifications) and country-level estimates.

Methods

Study design

The analysis was made based on the food balance sheets (FBS) compiled by FAO for 45 of the 54 African countries for the period 1990 to 2017 [13,14]. Data for the recent four years (2017-2020) had not been assembled and made available for public use and were not included in the analysis. Likewise, the data for Burundi, Comoros, Democratic Republic of Congo, Equatorial Guinea, Eritrea, Libyan, Seychelles, Somalia and Southern Sudan is not publicly available and the countries are not represented in the study.

The per capita supply of 95 major food commodities (kg/capita/day) was downloaded for each country-year. Then the energy, carbohydrate, fat, protein, SFA, MUFA, PUFA and cholesterol contents were determined by referring to a standard food composition database [15]. The per capita supply of fruits and vegetables was also computed.

Data source

Food Balance Sheet, also known as Food Disappearance data, is the compilation of the food supply of a country in a given period. FAO based on multiple data sources including official reports, determines the production, import, export, changes in stocks and non-food uses for major food commodities and estimates the food available for human consumption in a territory. FAO annually publishes the supply statistics of more than 90 primary (e.g. eggs, milk) and processed commodities (e.g. butter) for about 180 countries as the average of supply over the past three-year period [16]. Food Balance Sheet is an important tool for appraising food situations at various levels (global, regional or national) and informing national food and agriculture policies.

Estimation of nutrient composition

The supply of 95 commodities (kg/person/day) was converted into nutrient values (calorie, carbohydrate, protein, fat, SFA, MUFA, PUFA, sugar and cholesterol) using the US Department of Agriculture (USDA) Food Data Central food composition database [15]. At times when a food item is missing from the database, the FAO food composition table for international use was referred [16]. When a commodity aggregates two different foods (e.g. mutton and goat meat, oranges and mandarins, lemons and limes), the nutrient composition was estimated as the average of the two assuming equal weights. Similarly, when a group aggregates multiple food types (peas, beans, fresh water fish….) the specific food types within that domain were identified from the FAO FBS handbook [16], and the mean nutrient composition was determined assuming equal weights. Nutrient compositions for “others” (other cereals, other pulses, other oil crops, other meats etc...) were estimated by taking the arithmetic mean of the related commodities listed in the FBS.

The FBS provides all data on meat in terms of carcass weight that includes non-edible bones [16]. Carcase weight was converted into edible weight using the conversion factors recommended by USDA [17]. The conversion factor ranged from 70% for bovine meat to 100% for fish. For eggs conversion factor of 98.5 was also used [17].

The amount of all all energy-yielding macronutrients (carbohydrate, protein and fat) and alcohol available in the food supply for each country-year was converted into calories using Atwater specific factors [18]. Calories from various types of fatty acids (SFA, MUFA, PUFA) were also computed.

Estimation of energy contribution of free sugars

WHO defines "free sugars" as "*all monosaccharides and disaccharides added to foods by the manufacturer, cook or consumer, plus the sugars that are naturally present in honey, syrups and fruit juices*" [8,19]. In the current study, calories from free sugars were estimated by totalling the energy contents of honey and industrially produced sugars and sweeteners, with calories from monosaccharides and disaccharides contained in fruit juices. To the best of our knowledge, no information is available regarding what proportion of fruits are consumed as juice in Africa. Accordingly, based on the National Health and Nutrition Examination Survey (NHANES) of the US, we assumed that 35% of the total fruits were consumed as juice in all country-year [20].

Supply of fruits and vegetables

The supply of fruits and vegetables (g/capita/day) was estimated by summing the balances of all specific fruits and vegetables represented in the FBS. In line with the approach used by WHO [8] and other similar studies [21,22], starchy roots and tubers including cassava, yams, potato and sweet potato were not considered as vegetables.

Data management and analysis

The food supply statistics for each country-year was downloaded from the old (1990-2012) [13] and new (2013-2017) [14] FAOSTAT databases as csv files and exported to SPSS v24 for analysis. On top of regional (continental) and country-level figures, sub-regional estimates were computed by grouping countries based on geographic location and national income levels. The UN sub-region classification was used to classify countries into Northern, Eastern, Southern, Western and Central sub-regions [23]. Furthermore, the World Bank's

Gross National Income (GNI) classification was employed for stratifying countries as low-, lower-middle-, upper middle- or high-income economies [24]. The sub-regional and economic classification of the countries is provided as a supporting file (Supplementary File 1). Whenever estimates are provided by aggregating multiple countries, population-weighted analysis was used. Percentage change over the study period (1990-2017) was determined by dividing difference between the base- and end-year to the base value in 1990. PUFA to SFA ratio was computed by dividing the energy contribution of PUFA with that of SFA.

Results

Trends in energy supply

Between 1990 and 2017, the per capita supply of energy in Africa increased by 16.6% from 2,685 in 1990 to 3,132 kcal/person/day in 2017. The rates of increase were above the regional average in Central (28.8%) and Western (22.2%) sub-regions and in low-income countries (25.5%). The most recent figures from 2017 indicated that the energy supply (kcal/person/day) in low-income countries (2,771) was much lower than that of upper-middle- or high-income countries (3,448); furthermore, the supply was highest in Southern (3,406) and lowest in Eastern (2,625) sub-region.

Over the period, Africa had also seen significant improvements in the supply of all energy-yielding nutrients. Protein supply increased by 19.0%, whereas carbohydrate and fat supplies rose by 16.7 and 14.6%, respectively (Table 1).

Table 1: Trends in the supply of macronutrients in Africa, 1990-2017.

Supply of macronutrients	Year							% change
	1990	1995	2000	2005	2010	2015	2017	
Calorie supply (kcal/capita/day)								
Africa	2685	2791	2856	2942	3020	3144	3132	16.6
National income level								
Low-income	2208	2233	2355	2462	2562	2765	2771	25.5
Lower-middle-income	2907	3063	3106	3194	3271	3338	3312	13.9
Upper-middle- or high-income	3025	3026	3118	3182	3237	3402	3448	14.0
Sub-region								
Northern	2685	2791	2856	2942	3020	3144	3132	16.6
Central	2283	2320	2436	2628	2834	2940	2940	28.8
Southern	3004	2994	3087	3155	3201	3346	3406	13.4
Eastern	2267	2217	2286	2385	2436	2633	2625	15.8
Western	2700	3028	3102	3195	3261	3309	3300	22.2
Carbohydrate supply (g/capita/day)								
Africa	495.0	518.3	527.4	536.6	549.3	579.0	577.6	16.7
National income level								
Low-income	411.8	414.9	439.9	455.6	469.8	509.6	511.5	24.2
Lower-middle-income	538.6	575.0	576.7	585.8	600.0	620.9	616.7	14.5
Upper-middle- or high-income	513.4	510.0	526.0	518.4	518.5	562.8	574.4	11.9
Sub-region								
Northern	603.0	612.4	619.7	638.5	656.3	691.8	684.2	13.5
Central	403.9	420.2	431.2	465.2	506.6	513.0	521.0	29.0
Southern	513.4	508.9	524.7	517.7	516.9	556.3	570.3	11.1
Eastern	431.0	424.8	440.7	450.0	453.5	491.1	488.2	13.3
Western	488.1	560.1	566.5	571.4	589.8	615.2	615.7	26.1
Protein supply (g/capita/day)								
Africa	66.6	67.6	71.0	74.8	78.1	80.5	79.3	19.0
National income level								
Low-income	54.9	55.6	59.0	62.7	67.0	70.8	71.4	30.0
Lower-middle-income	71.0	72.3	76.2	80.3	83.7	85.1	82.9	16.8
Upper-middle- or high-income	83.9	82.7	85.1	88.0	88.4	90.2	89.6	6.8
Sub-region								
Northern	80.8	83.5	88.8	92.8	98.7	101.8	100.1	23.8
Central	57.6	55.3	62.2	66.5	72.9	83.5	80.3	39.4
Southern	82.9	81.2	83.6	86.8	86.9	88.0	87.9	6.0
Eastern	55.0	53.7	55.0	58.5	61.1	66.5	66.2	20.4
Western	65.1	68.6	73.1	77.8	81.1	79.2	77.9	19.7
Fat supply (g/capita/day)								
Africa	55.3	56.8	58.9	63.1	64.7	63.7	63.4	14.6
National income level								
Low-income	42.4	44.2	45.2	48.7	51.7	54.6	54.3	28.0
Lower-middle-income	60.2	61.2	64.2	68.7	69.4	66.3	65.9	9.5

Upper-middle- or high-income	73.6	77.4	79.9	88.9	94.7	91.6	92.1	25.2
Sub-region								
Northern	65.3	68.0	70.3	68.3	73.6	74.8	74.8	14.6
Central	53.0	51.9	57.0	61.3	62.8	66.0	64.5	21.6
Southern	72.0	75.1	77.9	86.9	92.2	89.5	90.2	25.2
Eastern	41.5	39.4	39.8	45.4	48.4	50.9	51.4	23.8
Western	58.0	62.1	66.0	72.9	70.4	64.5	63.6	9.6

Calorie contribution of energy-yielding macronutrients

Between 1990 and 2017, the contribution of carbohydrates to the total daily energy supply in Africa remained constant around 73%. In 2017, the carbohydrate's contribution was relatively lower in upper-middle- or high-income (65.6%) and Southern Africa (66.1%) countries but both were within the acceptable range of 55 to 75% (Table 2).

Country-specific figures indicated, in 2017 contribution of carbohydrate exceeded the upper limit of 75%, in seven countries including Madagascar (83.8%), Rwanda (78.9%), Ghana (78.9%), Mozambique (77.4%) and Nigeria (76.8%) (Supplementary File 2).

At regional-level, the calorie contribution of protein was around 9% and the figure remained below the minimum target of 10% in almost all sub-regions and income levels, with the exception of the Northern Africa sub-region (10.1%).

At regional-level, the calorie contribution of fat also remained constant around 10%. However, in 2017, the figure exceeded the limit of 20% in upper-middle- or high-income countries and in Southern Africa sub-region. Country-level estimates indicated, 21 countries including Sao Tome and Principe (35.3%), Gambia (28.0%), Mauritius (26.5%) and Tunisia (25.7%) exceeded the limit of 20% (Supplementary File 2).

Table 2: Contribution (%) of carbohydrate, protein and fat to total energy supply in Africa, 1990-2017.

Contribution to total calorie supply (%)	Year						
	1990	1995	2000	2005	2010	2015	2017
Carbohydrate contribution (%)							
Africa	72.9	73.6	73.2	72.2	72.0	73.0	73.2
National income level							
Low income	74.3	74.4	74.8	74.0	73.4	73.9	74.0
Lower-middle income	72.8	74.0	73.1	72.1	72.1	73.3	73.5
Upper-middle- or high-income	66.7	66.3	66.2	64.1	63.0	65.2	65.6
Sub-region							
Northern	72.4	71.8	71.3	72.0	71.2	71.9	71.9
Central	70.1	71.9	70.4	70.3	70.9	69.3	70.3
Southern	67.3	67.0	66.9	64.7	63.7	65.6	66.1
Eastern	75.3	76.3	76.7	75.0	74.0	74.3	74.1
Western	72.7	74.1	73.1	71.5	72.3	74.5	74.9
Protein contribution (%)							
Africa	8.9	8.6	8.8	9.0	9.2	9.1	8.9
National income level							
Low income	8.9	8.7	8.8	9.0	9.2	9.0	9.1
Lower-middle-income	8.8	8.4	8.7	8.9	9.1	9.0	8.8
Upper-middle- or high-income	9.8	9.6	9.6	9.8	9.8	9.5	9.3
Sub-region							
Northern	9.5	9.7	9.9	10.2	10.4	10.2	10.1
Central	9.2	8.3	8.9	8.9	9.0	10.2	9.7
Southern	9.7	9.5	9.5	9.7	9.6	9.4	9.2
Eastern	8.6	8.4	8.3	8.5	8.7	8.8	8.8
Western	8.4	7.9	8.3	8.6	8.7	8.3	8.1
Total fat contribution (%)							
Africa	17.9	17.6	17.8	18.5	18.6	17.6	17.5
National income level							
Low-income	16.7	16.7	16.3	16.9	17.3	16.9	16.7
Lower-middle-income	18.2	17.4	18.0	18.8	18.6	17.3	17.4
Upper-middle- or high-income	21.3	22.4	22.5	24.5	25.7	23.7	23.5
Sub-region							
Northern	18.0	18.5	18.8	17.8	18.4	17.9	18.0
Central	20.3	19.4	20.2	20.3	19.2	19.6	19.2
Southern	21.0	21.9	22.1	24.1	25.2	23.4	23.2
Eastern	15.9	15.2	14.9	16.2	17.1	16.5	16.7
Western	18.7	17.9	18.5	19.8	18.8	16.9	16.7

Energy contribution of specific types of fatty acids

Table 3 shows the trends in per capita supply of SFA, MUFA and PUFA, expressed as contribution to total energy. In Africa, the calorie contribution of the fatty acids is found to be balanced. In 2017, 5.1% of the total calories came from UFA whereas, MUFA and PUFA contributed for 5.7 and 5.3%, respectively. Over the period (1990-2017) meaningful changes have not been seen at regional or sub-regional levels regarding the energy derived from the three groups of fatty acids (Table 3).

In 2017, the energy from SFAs remained within the acceptable range (<10%) in all countries except in Sao Tome and Principe (26.0%). The supply of PUFAs, on the other hand was sub-optimal (< 6%) in 28 African countries including Madagascar (2.0%), Liberia (3.0%), Sierra Leone (3.0%), Ghana (3.0%) and Rwanda (3.1%) (Supplementary File 2).

The balance between SFA and PUFA in a diet can also be measured using PUFA to SFA ratio. Over the period, the ratio remained around 1:1 in Africa. In 2017, the ratio was relatively higher in upper-middle- or high-income countries (1.54:1) and in Southern (1.56:2) and Northern (1.56:1) sub-regions suggesting the dominance of PUFA. Conversely, the ratio was low (0.76:1) in the Western sub-region indicating the opposite.

Table 3: Calorie contribution (%) of different groups of fatty acids to the total energy supply in Africa, 1990-2017.

Contribution to total calorie (%)	Year						
	1990	1995	2000	2005	2010	2015	2017
Saturated fatty acids contribution (%)							
Africa	5.4	5.2	5.1	5.7	5.7	5.1	5.1
National income level							
Low income	4.9	4.9	4.6	5.0	5.2	4.8	4.8
Lower-middle income	5.6	5.3	5.4	6.0	5.9	5.2	5.3
Upper-middle or high income	5.5	5.4	5.5	6.1	6.3	5.8	5.6
Sub-region							
Northern	4.9	5.0	5.2	5.2	5.2	4.6	4.6
Central	6.1	5.6	5.6	5.6	5.3	5.2	5.1
Southern	5.4	5.3	5.4	6.0	6.2	5.7	5.5
Eastern	4.8	4.5	4.3	4.9	5.4	4.8	4.9
Western	6.2	6.0	5.8	6.6	6.2	5.6	5.5
Monounsaturated fatty acids contribution (%)							
Africa	5.6	5.5	5.6	5.8	5.9	5.7	5.7
National income level							
Low-income	5.4	5.4	5.3	5.4	5.5	5.6	5.5
Lower-middle-income	5.7	5.4	5.7	5.9	5.9	5.6	5.6
Upper-middle- or high-income	6.3	6.5	6.7	7.3	7.8	7.4	7.4
Sub-region							
Northern	5.5	5.7	5.9	5.3	5.6	5.7	5.8
Central	7.0	6.8	7.2	7.3	6.9	7.2	6.9
Southern	6.2	6.3	6.5	7.1	7.6	7.3	7.2
Eastern	4.9	4.5	4.5	4.9	5.2	5.1	5.1
Western	6.2	5.9	6.2	6.4	6.2	5.6	5.6
Polyunsaturated fatty acids contribution (%)							
Africa	5.3	5.3	5.4	5.5	5.4	5.2	5.3
National income level							
Low income	4.8	4.9	4.8	4.9	4.9	4.9	4.9
Lower-middle income	5.4	5.2	5.4	5.4	5.3	5.1	5.1
Upper-middle or high income	7.6	8.6	8.4	9.2	9.6	8.7	8.6
Sub-region							
Northern	6.3	6.4	6.2	5.7	6.0	6.1	6.2
Central	5.5	5.4	5.8	5.7	5.4	5.4	5.4
Southern	7.4	8.4	8.3	9.1	9.5	8.6	8.6
Eastern	4.7	4.6	4.6	4.9	4.9	5.1	5.1
Western	4.7	4.5	4.9	5.1	4.8	4.3	4.2
Polyunsaturated to saturated fatty acid ratio							
Africa	0.98	1.02	1.06	0.96	0.95	1.02	1.04
National income level							
Low-income	0.98	1.00	1.04	0.98	0.94	1.02	1.02
Lower-middle-income	0.96	0.98	1.00	0.90	0.90	0.98	0.96

Upper-middle- or high-income	1.38	1.59	1.53	1.51	1.52	1.50	1.54
Sub-region							
Northern	1.29	1.28	1.19	1.10	1.15	1.33	1.35
Central	0.90	0.96	1.04	1.02	1.02	1.04	1.06
Southern	1.37	1.58	1.54	1.52	1.53	1.51	1.56
Eastern	0.98	1.02	1.07	1.00	0.91	1.06	1.04
Western	0.76	0.75	0.84	0.77	0.77	0.77	0.76

Energy contribution of free sugars

Over the period, the energy contribution of free sugars (%) in Africa remained constant around 7%. In 2017, the figure exceeded the upper limit of 10% in upper-middle- or high-income countries (14.7%) and in Southern (14.8%) and Northern (10.5%) sub-regions. At country-level, thirteen countries exceed the limit including Botswana (18.8%), Namibia (16.4%), Eswatini (15.2%) and South Africa (14.9%) (Supplementary File 3) (Table 4).

Table 4: Contribution of free sugars (%) to the total calorie supply in Africa, 1990-2017.

Contribution of free sugars to the total calorie (%)	Year						
	1990	1995	2000	2005	2010	2015	2017
Africa	6.9	6.8	7.0	7.0	7.1	7.0	7.1
National income level							
Low-income	5.2	5.2	5.2	5.9	5.8	6.0	6.0
Lower-middle-income	7.2	7.1	7.5	7.4	7.5	7.0	6.9
Upper-middle- or high-income	12.8	11.6	10.9	10.2	11.2	14.0	14.7
Sub-region							
Northern	10.2	9.5	10.3	10.6	10.8	10.5	10.5
Central	6.1	5.3	5.3	5.9	5.8	5.8	6.4
Southern	12.7	11.6	10.9	10.2	11.3	14.1	14.8
Eastern	5.6	5.9	6.3	6.1	6.2	6.2	6.2
Western	4.4	4.9	4.7	5.1	5.1	4.7	4.6

Cholesterol supply

At regional-level the total dietary cholesterol supply increased by 14% from 92.8 in 1990 to 105.7 mg/capita/day in 2017. The increase was also observed almost in all economic levels and sub-regions. Despite the progressive rise, cholesterol supply remained within the tolerable range set by WHO (<300 mg/day). Country-level estimates are given in a supplementary file (Supplementary File 4) (Table 5).

Table 5: Dietary cholesterol supply (mg/capita/day) in Africa, 1990-2017.

Dietary cholesterol supply (mg/capita/day)	Year							% change
	1990	1995	2000	2005	2010	2015	2017	
Africa	92.8	88.5	93.1	101.4	111.7	110.5	105.7	14.0
National income level								
Low income	58.8	55.0	57.4	63.0	68.3	65.5	65.0	10.7
Lower-middle income	99.1	93.9	100.7	110.1	122.0	120.3	114.4	15.4
Upper-middle or high income	196.0	194.1	200.3	223.7	258.0	274.4	260.2	32.8
Sub-region								
Northern	119.9	123.9	141.1	153.1	173.6	183.0	177.5	48.1
Central	91.1	74.8	83.6	87.8	101.8	127.8	116.6	28.0
Southern	187.6	184.4	189.8	213.8	246.1	263.7	250.5	33.5
Eastern	60.5	51.2	49.3	54.1	59.4	57.8	56.6	-6.4
Western	82.2	78.8	82.5	92.6	99.5	87.1	83.3	1.4

Fruits and vegetables supply

Over the period (1990-2017) the availability of fruits and vegetables per person in Africa rose by 27.5% from 279 to 356 g/capita/day. The trends in the supply of fruits and vegetables in different sub-regions and economic levels are shown in Fig 1 and 2. In terms of economic levels, the supply increased by 37.0 and 13.2% in lower-middle- and low-income countries, respectively, but had fallen by 20.8% in upper-middle- or high-income countries. Regarding geographical sub-regions, the supply substantially increased by 57.7% in Northern Africa. Considerable improvements have been seen in Western (33.3%) and Central (28.0%) sub-regions as well. Conversely, the improvements were modest (5.9%) in Eastern sub-region and

even a considerable decline (22.6%) was seen in Southern sub-region. In general, the supply of fruits and vegetables remained below ~~below~~ the minimum target of 400 g/capita/day in all regions and economic levels except in Northern Africa and lower-middle-income countries. Country-level estimates are given as a supplementary file (Supplementary File 5).

Fig 1. Trends in the supply of fruits and vegetables in sub-regions of Africa, 1990-2017.

Fig 2. Trends in the supply of fruits and vegetables across national income levels in Africa, 1990-2017.

Discussion

The purpose of the current study was to evaluate the African food supply against population nutrient intake goals set for preventing diet-related NCDs, and provide regional, sub-regional and country-level estimates.

According to the recommendation of WHO, the fraction of energy derived from fat should not exceed 35% of the total intake and threshold of 20% is more compatible with good health [8]. This analysis suggested, between 1990 and 2017 the energy contribution of fat in Africa did not meaningfully change and in 2017 fat provided less than 35% of energy at all levels (regional, sub-regional or country). Yet, the figure exceeded 20% in 21 countries. Though the finding is potentially worrying, it is less alarming than what is being observed in the other regions of the world. According to an estimate, in 2013 Africa had the lowest (54.5 g/capita/day) fat supply, and between 1961 and 2013 the supply was only increased by 15 g/capita/day. Conversely, fat supply markedly increased by 52, 48, 30 g/capita/day respectively, in South America, Asia and the Caribbean regions [25].

From the perspective of preventing both under- and over-nutrition, protein should contribute 10-15% of the daily energy intake [8]. However, the current analysis indicated that in 2017 energy derived from protein remained below 10% in all sub-regions except in Northern Africa. The finding indicates that the existing food supply in the region favours protein undernutrition that overnutrition. A global estimate indicated, between 1961 and 2013 the protein supply per person in Africa was only increased by 31%, which was much lower than advances made in Asia (64%) and the Caribbean (46%) regions [25]. The supply in Africa (69.10 g/capita/day) was also the lowest among all other regions of the world [25].

Diet should contain an optimal amount of PUFAs, 6-10% of the total energy. PUFAs reduce plasma LDL and lessen the risk of cardiovascular diseases [8,26]. Apart from NCDs, ω-3 PUFAs are also required for brain development during early stages of life [27]. In Africa, with the exception of the Southern and Northern sub-regions, the PUFA supply appears to be suboptimal (<6% of energy). Very few studies have so far evaluated the adequacy of PUFA supply at national or international levels [26,28-30]. According to an estimate, globally only 1.3% of the total energy comes from PUFA, while MUFA and UFA, contribute 6.4% and 6.7%, respectively indicating suboptimal supply of PUFA is a global concern [26].

Generally, it is assumed that the desirable ratio of PUFA to SFA in the diet is 1:1 [31,32]. In the current study, the ratio was around the optimal level at regional level but it was much ~~low~~ in the Western sub-region (0.76:1) suggesting the dominance of SFAs. From NCDs perspective this is worrying because SFAs have strong tendency to increase LDL ("bad cholesterol") [8]. Analysis of national FBS data of Ethiopia [30] and Trinidad and Tobago [29] also indicated that the PUFA to SFA ratios were lower than one indicating the dominance of SFA in the respective national food supplies.

High dietary sugar intake causes positive energy balance and ultimately leads to NCDs through encouraging unhealthy weight gain [8]. It is recommended that the contribution of free sugars should not exceed 10% of the total energy [8] and further reduction to 5% provides additional benefits [19]. While free sugar intake in Africa remains within the acceptable range, the figure exceeded 10% in many ~~counties,~~ especially those in Southern and Northern sub-regions. This may partly explain the higher ~~significance~~ of NCDs in these sub-regions [5]. According to WHO, percentage of deaths from NCDs in Southern and Northern sub-regions is 70%, as compared to 41% in the entire Africa [5]. A study also

indicated, between 1961 and 2007, calorie derived from sugars had substantially increased in Northern Africa by 63% [33].

We estimated the energy contribution of free sugars by assuming that 35% of fruits were consumed as juice in all country-years. The assumption was made based on the finding of a national survey conducted in US [20]. This decision might have made us to over or under-estimate the intake of free sugars because the consumption pattern of whole fruits is likely to vary among different regions and countries. It can also ~~be~~ change over time. Furthermore, as the consumption of sugars considerably ~~vary~~ across different stages of lifespan [34], the per capita supply statistics may not adequately capture the inter-individual variation in intake.

Even though the effect of dietary cholesterol on serum cholesterol has long been debated [35-37], the existing WHO's nutrient intake recommends for restricting intake below 300 mg/day [8]. According to our finding, between 1990 and 2017, the total dietary cholesterol supply in Africa has increased by 14% but it remained below the aforementioned threshold in all sub-regions and economic levels. Very few studies have so far explored the supply of dietary cholesterol at national-level [29,38]. In Trinidad and Tobago, between 1961 and 2007, the supply increased by 80% to 225 mg/day [29]; whereas the corresponding level in Finland was 440 mg/day [38].

Adequate intake of fruits and vegetables contributes to the reduction of energy density, promotes the consumption of dietary fibre, and reduces the risk of NCDs including obesity, cardiovascular diseases and possibly gastrointestinal cancers [8]. The current analysis suggested that, in Africa the fruits and vegetables supply is gradually improving but remains below the minimum target of 400 g/person/day in all sub-regions, except in the North. A previous study estimated that in 2005 the fruit and vegetable availability was around 546 g/person/day globally, and the lowest supply was in sub-Saharan Africa (206 g/person/day)

and South Asia (326 g/person/day). Conversely, the middle east and north Africa had one of the highest supplies (735 g/person/day) [22]. A multicounty study that enrolled 10 sub-Saharan Africa countries concluded that in all countries the per capita consumption of fruits and vegetables was below 50g/day [27]. In addition to NCDs, low intake of fruits and vegetables had been identified as a major driver of micronutrient deficiencies in Africa [39].

This study evaluated the African food supply against multiple population intake goals set for prevent NCDs. However, the following limitations should be taken into consideration while interpreting the findings. The overall analysis is made by considering food supply as a proxy indicator of food consumption; however, this assumption is not strictly true and might have cause overestimation of the intake of the nutrients. While FBS is a useful tool for international comparison and analysis over time, it does not take within country variation including geographic, seasonal and interpersonal differences, into account [16]. Furthermore, FBS does not take household-level food wastage and subsistent production of less pertinent food commodities, into consideration and this might have caused overestimation of intake.

On top of the inherent limitations of FBS, we did not evaluate the African food supply against nutrient intake goals set for ω-3 and ω -6 fatty acids because a comprehensive food composition database is not available for these nutrients. Further, intake goals set for TFAs and fibres had not been evaluated because naturally occurring trans fats are less relevant to NCDs than their artificial counterparts [40]. Similarly, the intake of fibres is more affected by food processing rather than total food supply. Irrespective of these methodological shortcomings, the study has provided comprehensive information on the African food supply from NCDs perspective and permitted ~~for~~ between-country and over time comparisons.

Conclusion

Between 1990 and 2017, the per capita supply of calorie substantially increased in Africa, including all sub-regions and economic categories. Most population intake goals set for preventing NCDs remained within acceptable range. However, the supplies of fruits and vegetables and PUFAs are low and the increasing energy contributions of free sugars and fats are emerging concerns in specific sub-regions and countries of Africa.

Acknowledgment

The author acknowledges the Food and Agriculture Organization of the UN for making the food balance sheets data publicly available without restrictions.

References

1. WHO. Noncommunicable disease: fact sheet. 2017. [Cited 2020, October 05] Available from: http://www.who.int/mediacentre/factsheets/fs355/en/.
2. Martinez R, Lloyd-Sherlock P, Soliz P, Ebrahim S, Vega E, Ordunez P, *et al*. Trends in premature avertable mortality from non-communicable diseases for 195 countries and territories, 1990–2017: a population-based study. Lancet Glob Health. 2020; 8: e511–23. doi: 10.1016/S2214-109X(20)30035-8.
3. NCD Countdown 2030 collaborators. NCD Countdown 2030: Pathways to achieving Sustainable Development Goal target 3.4. Lancet. 2020; 396: 918–34. doi: 10.1016/S0140-6736(20)31761-X.
4. Dain K. Challenges Facing Global Health Networks: The NCD Alliance Experience, comment on "Four Challenges that Global Health Networks Face". Int J Health Policy. Manag. 2018; 7(3): 282–285. doi: 10.15171/ijhpm.2017.93.
5. WHO. Noncommunicable diseases: Progress monitor 2020. Geneva: World Health Organization; 2020.
6. Gouda HN, Charlson F, Sorsdahl K, Ahmadzada S, Ferrari AJ, Erskine H, *et al*. Burden of non-communicable diseases in sub-Saharan Africa, 1990–2017: Results from the Global Burden of Disease Study 2017. Lancet Glob Health. 2019; 7: e1375–87. doi:1016/S2214-109X(19)30374-2.
7. GBD Diet Collaborators. Health effects of dietary risks in 195 countries, 1990–2017. Lancet. 2019; 393: 1958–72. doi: 1016/S0140-6736(19)30041-8.
8. WHO. Diet, nutrition and the prevention of chronic diseases. Report of a Joint WHO/FAO Expert Consultation. Geneva: World Health Organization; 2003.

9. Branca F, Lartey A, Oenema S, Aguayo V, Stordalen GA, Richardson R, *et al*. Transforming the food system to fight non-communicable diseases. BMJ. 2019 364: l296. doi: 10.1136/bmj.l296.
10. Micha R, Shulkin ML, Peñalvo JL, Khatibzadeh S, Singh GM, Rao M, *et al*. Etiologic effects and optimal intakes of foods and nutrients for risk of cardiovascular diseases and diabetes: systematic reviews and meta-analyses from the Nutrition and Chronic Diseases Expert Group (NutriCoDE). PLoS One. 2017; 12: e0175149. doi: 0.1371/journal.pone.0175149.
11. Food and Agriculture Organization of the United Nations (FAO). Fats and fatty acids in human nutrition: Report of an expert consultation. Rome: FAO; 2010.
12. Smit LA, Mozaffarian D, Willett W. Review of fat and fatty acid requirements and criteria for developing dietary guidelines. Ann Nutr Metab. 2009; 55:44-55. doi: 10.1159/000228995.
13. FAO. FAOSTAT: Food Balances (old methodology and population). 2019. [Cited 2020, October 05] Available from: http://www.fao.org/faostat/en/#data/FBSH.
14. FAO. FAOSTAT: New Food Balances. 2019. [Cited 2020, October 05] Available from: http://www.fao.org/faostat/en/#data/FBS.
15. US Department of Agriculture (USDA). FoodData Central. [Cited 2020, October 05] Available from: https://fdc.nal.usda.gov/.
16. FAO. Food balance sheets: A hand book. Rome: FAO; 2001.
17. Putnam JJ, Allshouse JE. Food consumption, prices, and expenditures, 1970-97: Statistical Bulletin No. 965.Washington, DC: Food and Rural Economics Division, Economic Research Service, U.S. Department of Agriculture; 1999.
18. FAO. Food energy – methods of analysis and conversion factors: FAO food and nutrition paper 77. Rome: FAO; 2003.

19. WHO. Information note about intake of sugars recommended in the WHO guideline for adults and children. Geneva: WHO; 2015.

20. Drewnowski A, Rehm CD. Socioeconomic gradient in consumption of whole fruit and 100% fruit juice among US children and adults. Nutr J. 2015; 14:3. doi:10.1186/1475-2891-14-3.

21. Herforth A, Arimond M, Álvarez-Sánchez C, Coates J, Christianson K, Muehlhoff E. A global review of food-based dietary guidelines. Adv Nutr. 2019; 10(4): 590–605. doi: 10.1093/advances/nmy130.

22. Mason-D'Croz D, Bogard JR, Cenacchi N, Dunston S, Herrero M, Wiebe K. Gaps between fruit and vegetable production, demand, and recommended consumption at global and national levels: an integrated modelling study. Lancet Planet Health. 2019; 3(7): e318-e329. doi: 10.1016/S2542-5196(19)30095-6.

23. United Nations Statistics Division. Standard country or area codes for statistical use. 2020. [Cited 2020, October 05] Available from: https://unstats.un.org/unsd/methodology/m49/.

24. World Bank. Country and lending groups: Country classification. 2020. [Cited 2020, October 05] Available from: https://datahelpdesk.worldbank.org/knowledgebase/articles/906519-world-bank-country-and-lending-groups.

25. Roser M, Ritchie H. Our world in data: Food Supply. 2013. [Cited 2020, October 05] Available from: https://ourworldindata.org/food-supply.

26. Schmidhuber J, Sur JP, Fay K, Huntley B, Salama J, Lee A, *et al*. The Global Nutrient Database: availability of macronutrients and micronutrients in 195 countries from 1980 to 2013. Lancet Planet Health. 2018; 2: e353–68. doi: 10.1016/S2542-5196(18)30170-0.

27. Innis SM. Dietary omega 3 fatty acids and the developing brain. Brain Res. 2008; 1237: 35-43. doi: 10.1016/j.brainres.2008.08.078.

28. Sanders T. Polyunsaturated fatty acids in the food chain in Europe. Am J Clin Nutr. 2002; 71(Suppl): S176-S178. doi: 10.1093/ajcn/71.1.176s.

29. Sheehy T, Sharma S. Trends in energy and nutrient supply in Trinidad and Tobago from 1961 to 2007 using FAO food balance sheets. Public Health Nutr. 2013;16(9):1693-702. doi: 10.1017/S136898001200537X.

30. Sheehy T, Carey E, Sharma S, Biadgilign S. Trends in energy and nutrient supply in Ethiopia: a perspective from FAO food balance sheets. Nutr J. 2019; 18:46. doi: 10.1186/s12937-019-0471-1.

31. Hayes KC. Dietary fat and heart health: in search of the ideal fat. Asia Pacific J Clin Nutr. 2002; 11 (Suppl): S394-S400. doi: 10.1046/j.1440-6047.11.s.7.13.x.

32. Grundy SM. What is the desirable ratio of saturated, polyunsaturated, and monounsaturated fatty acids in the diet? Am J Clin Nutr. 1997; 66(suppl): S988-S990.

33. Golzarand M, Mirmiran P, Jessri M, Toolabi K, Mojarrad M, Azizi F. Dietary trends in the Middle East and North Africa: An ecological study (1961 to 2007). Public Health Nutr. 2012; 15(10): 1835–1844. doi: 10.1017/S1368980011003673.

34. Newens KJ, Walton J. A review of sugar consumption from nationally representative dietary surveys across the world. J Hum Nutr Diet. 2016 Apr;29(2):225-40. doi: 10.1111/jhn.12338.

35. Hopkins PN. Effects of dietary cholesterol on serum cholesterol: a meta-analysis and review. Am J Clin Nutr. 1992; 55:1060-1070. doi: 10.1093/ajcn/55.6.1060.

36. Soliman GA. Dietary cholesterol and the lack of evidence in cardiovascular disease. Nutrients. 2018; 10(6): 780. doi: 10.3390/nu10060780.

37. Berger S, Raman G, Vishwanathan R, Jacques PF, Johnson EJ. Dietary cholesterol and cardiovascular disease: a systematic review and meta-analysis. Am J Clin Nutr. 2015;102(2):276-94. doi: 10.3945/ajcn.114.100305.

38. Heinonen M, Lampi AM, Hyvonen L, Homer D. The fatty acid and cholesterol content of the average Finnish diet. J Food Compost Anal.1992; 5(3):198-208. doi: 10.1016/0889-1575(92)90038-l.

39. Ruel MT, Minot N, Smith L. Patterns and comparison of fruits and vegetables consumption in sub-Saharan Africa: a multicounty comparison. Geneva: WHO; 2005.

40. U.S. Department of Agriculture and U.S. Department of Health and Human Services. Dietary Guidelines for Americans. 7th Edition, Washington, DC: U.S. Government Printing Office; 2010.

Supporting information captions

Supplementary file 1: Sub-regional and economic classification of the 45 countries included in the study.

Supplementary file 2: Country-specific estimates: Contribution (%) of carbohydrate, protein and fat to total energy supply in Africa, 1990-2017.

Supplementary file 3: Country-specific estimates: Contribution of free sugars (%) to the total calorie supply in Africa, 1990-2017.

Supplementary file 4: Country-specific estimates: Dietary cholesterol supply in Africa, 1990-2017.

Supplementary file 5: Country-specific estimates: Fruit and vegetable supply in Africa, 1990-2017.

Figure 1

Click here to access/download;Figure;Fig 1.tif

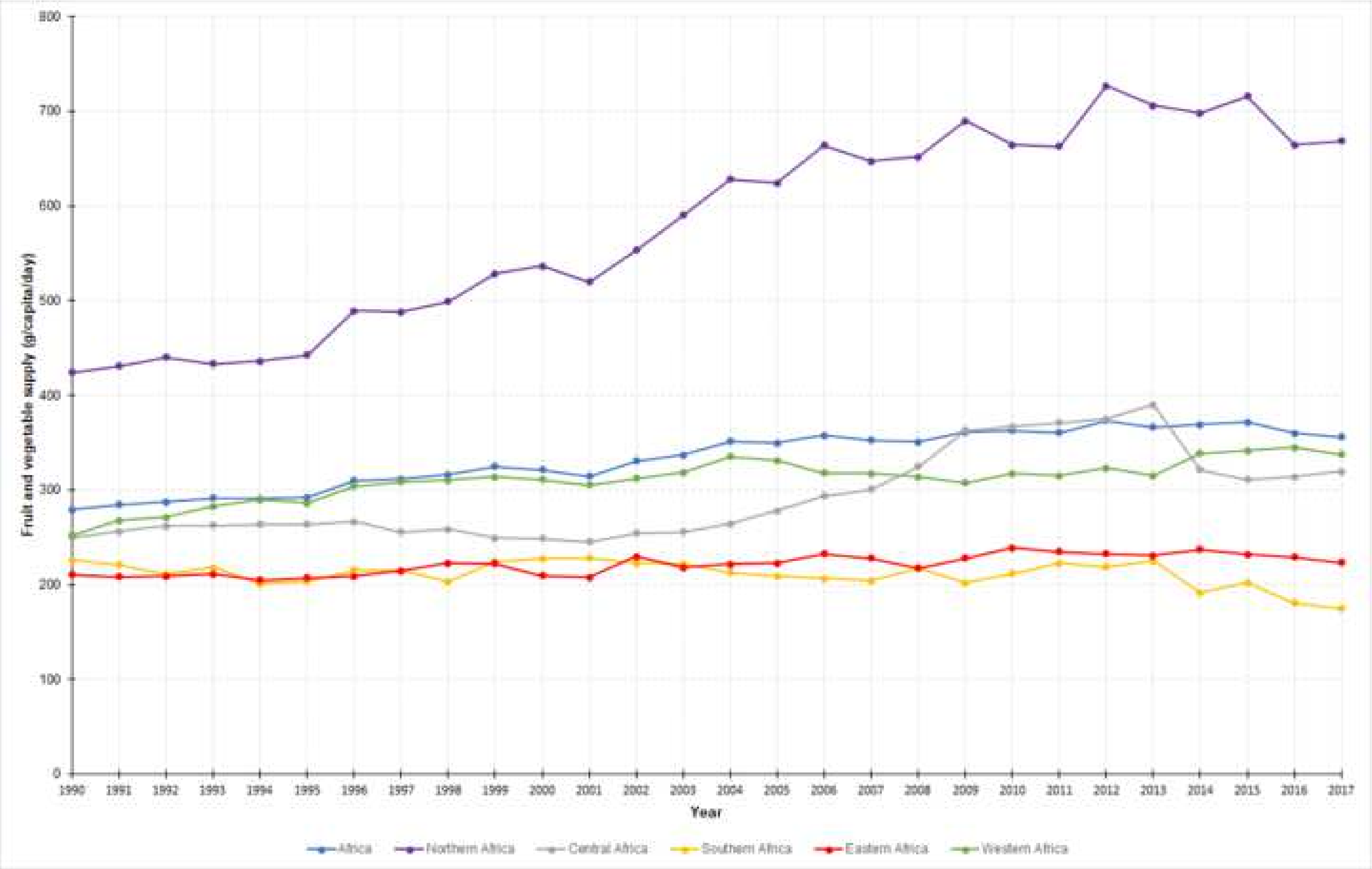

Figure 2

Click here to access/download;Figure;Fig 2.tif

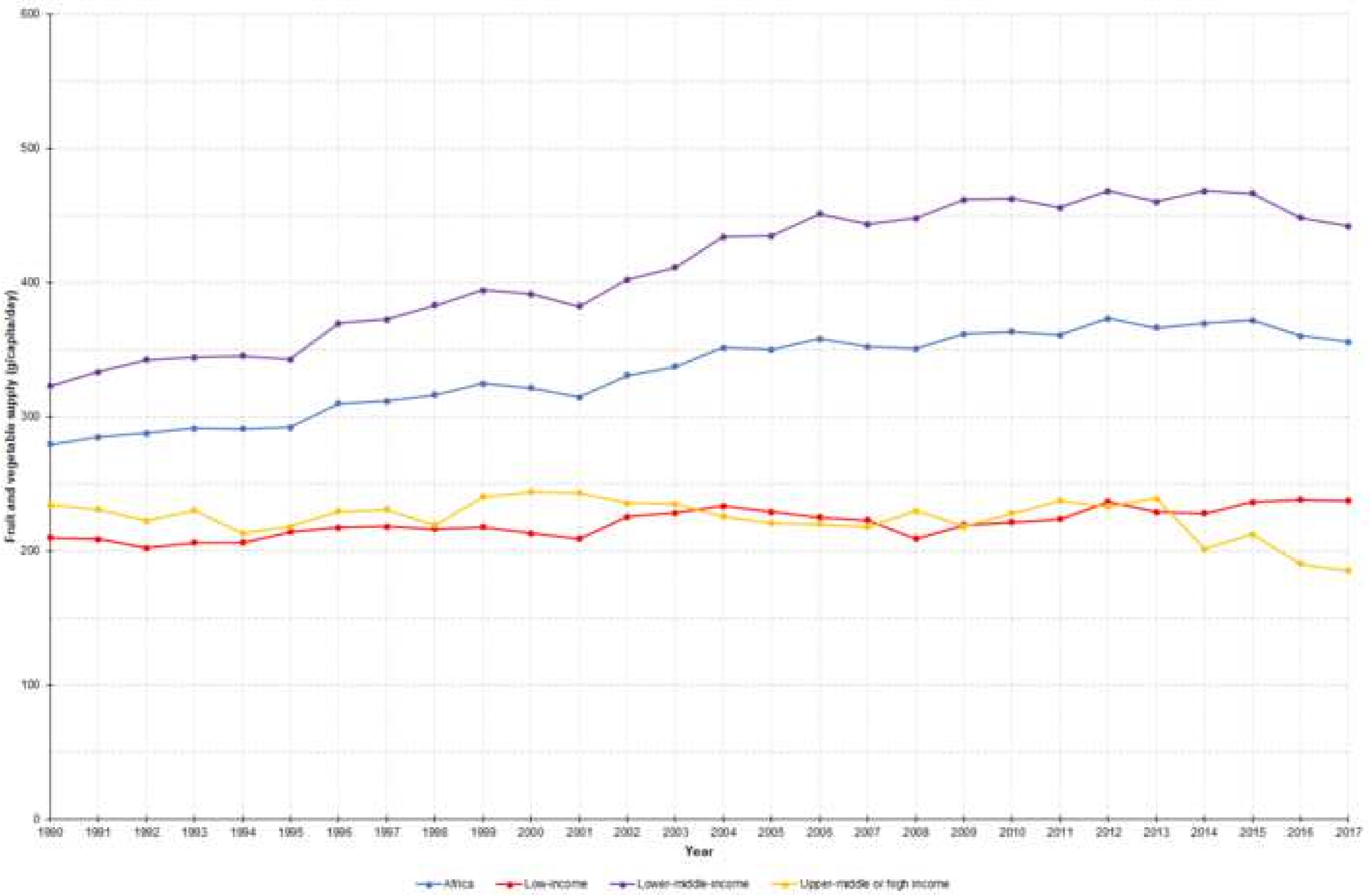

Supplementary file 1

Click here to access/download
Supporting Information
Supplementary File 1.xlsx

Supplementary file 2

Click here to access/download
Supporting Information
Supplementary File 2.xlsx

Supplementary file 3

Click here to access/download
Supporting Information
Supplementary File 3.xlsx

Supplementary file 4

Click here to access/download
Supporting Information
Supplementary File 4.xlsx

Supplementary file 5

Click here to access/download
Supporting Information
Supplementary File 5.xlsx
